# Successful Management of Rhino-Orbital Mucormycosis in a Diabetic Patient: A Case Report

**DOI:** 10.7759/cureus.76739

**Published:** 2025-01-01

**Authors:** Stanislav Tsisar, Mariana Salvado de Morais, Rita Gameiro, Mario Rodrigues

**Affiliations:** 1 Internal Medicine, Centro Hospitalar Universitário Lisboa Central - Hospital de São José, Lisbon, PRT

**Keywords:** diabetes mellitus, fungal infection, mucormycosis, rhino-orbital mucormycosis, rhinosinusitis

## Abstract

Mucormycosis is a rare but aggressive fungal infection, primarily affecting immunocompromised patients, with diabetes being a significant risk factor. This report describes the case of a 20-year-old male with poorly controlled type 1 diabetes who presented with facial swelling, proptosis, and necrotic nasal lesions. Imaging and biopsy confirmed rhino-orbital mucormycosis with* Rhizopus arrhizus*. The patient underwent multiple surgical debridements and received dual antifungal therapy with liposomal amphotericin B and isavuconazole, alongside adjunctive hyperbaric oxygen therapy. Despite multiple complications, such as septic and cardiogenic shock, "in and out" diabetic ketoacidosis, and long-term oral compromise, clinical stabilization was achieved after prolonged hospitalization and a multidisciplinary approach. Currently, the patient is clinically and radiologically stable over two years of suppressive therapy with isavuconazole. This case highlights the importance of early diagnosis, aggressive multidisciplinary management, and tailored antifungal therapy in the treatment of rhino-orbital mucormycosis.

## Introduction

Mucormycosis, an aggressive and often fatal fungal infection caused by molds of the order Mucorales, has emerged as a significant concern in immunocompromised populations [1]. Its most common clinical forms include rhino-orbital-cerebral, pulmonary, gastrointestinal, cutaneous, and disseminated mucormycosis, with the rhino-orbital-cerebral form being particularly devastating. These fungi, typically found in soil, decaying organic matter, and dust, rarely cause disease in healthy individuals. However, in patients with underlying conditions such as uncontrolled diabetes, malignancies, or recent organ transplants, mucormycosis can lead to severe morbidity and mortality [1]. The hyperglycemic environment in diabetic ketoacidosis further promotes fungal proliferation, making early diagnosis and timely intervention critical [2]. The annual incidence of the disease is 1.7 cases per 1,000,000 inhabitants [3]. This report details the clinical course of a 20-year-old male with poorly controlled diabetes, presenting with rhino-orbital mucormycosis. The case highlights diagnostic challenges, therapeutic approaches, and the multidisciplinary efforts required to manage this life-threatening condition effectively.

## Case presentation

**Figure 1:**
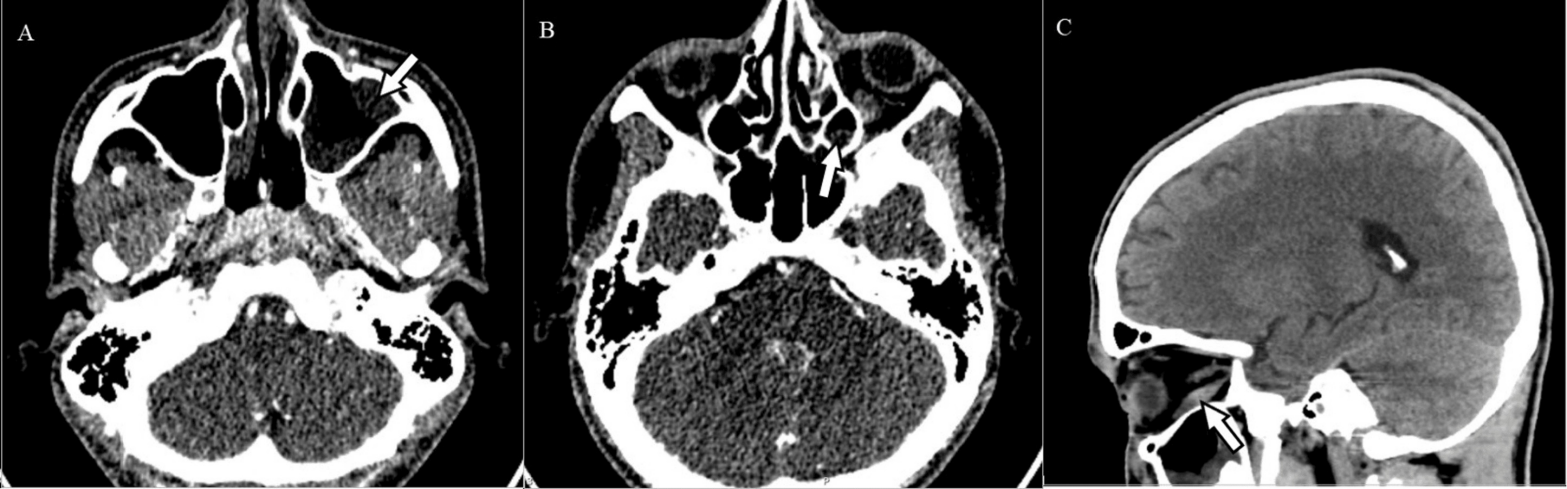
Head computed tomography scan at presentation The arrow shows (A) diffuse inflammation of left maxillary sinus mucosa, (B) inflammation of ethmoid sinus mucosa, and (C) diffuse densification of left intraorbital adipose tissue and increased thickness of homolateral inferior rectus muscle. All signs indicate an active rhino-orbital infection.

A 20-year-old male with a longstanding history of poorly controlled type 1 diabetes (HbA1c 9.7% at admission) was referred to our center presenting with facial swelling, proptosis, and blurred vision in his left eye. The patient reported worsening facial pain and edema for a month, accompanied by nasal obstruction, purulent rhinorrhea, and epistaxis. Despite multiple visits to healthcare providers and a diagnosis of odontogenic sinusitis, his condition continued to deteriorate. On physical examination, he presented with left periorbital edema, ptosis, complete ophthalmoparesis of the left eye, and an absent pupillary light reflex. A nasoendoscopy showed extensive necrotic plaques on the septal nasal mucosa of the right nasal cavity and on the floor and lateral wall of the left nasal cavity, along with purulent discharge from the middle meatus. A CT scan revealed diffuse densification of the left intraorbital adipose tissue and increased thickness of the homolateral inferior rectus muscle (Figure 1). An active inflammatory process in the frontal, maxillary, and sphenoid sinuses was observed. There was no intracranial involvement, with patent cavernous sinuses and ophthalmic vasculature. A severe rhino-orbital infection of probable fungal etiology was assumed, and the patient underwent emergent surgical intervention. A debridement of nasal mucosa and perinasal sinuses with the removal of necrotic tissue was performed. Empiric treatment was initiated with ceftriaxone, metronidazole, and liposomal amphotericin B at 10 mg/kg/day. In the first 24 hours, another surgical procedure was performed, with extensive debridement of the left maxillary and ethmoidal sinuses, partial removal of the hard palate and nasal septum, and complete removal of the left inferior conchae. The microscopic examination of surgical tissue showed hyphae attributable to Mucorales fungi and gram-negative bacilliform bacterial colonies. Later growth on microbiological examinations showed a *Rhizopus arrhizus* complex. *Pseudomonas aeruginosa *was also isolated and treated with a cycle of ceftazidime. After a multidisciplinary discussion with otolaryngologists, ophthalmologists, and maxillofacial surgeons, hyperbaric oxygen sessions were added to the treatment regimen.

The postoperative period was monitored in an intensive care unit. While there, the patient developed septic and cardiogenic shock due to *Klebsiella pneumoniae *bacteremia originating from a central venous catheter. There was multiorgan failure, and the patient required orotracheal intubation and mechanical ventilation, vasopressor, and inotropic support with norepinephrine and dobutamine, as well as transient hemodialysis. At that time, despite no progression of rhinosinusitis, isavuconazole was added to antifungal therapy, given the severity of the presentation. Later, the patient was downgraded to an intermediate unit where the main problem was diabetic ketoacidosis, especially given the oral intolerance caused by amphotericin. After discussion with gastroenterology, a percutaneous endoscopic gastrostomy was placed, and multiple antiemetic medications were added. The patient also transitioned to basal-bolus insulin for glycemic control and maintained the established treatment plan for mucormycosis. He underwent another surgical intervention with debridement of the necrotic tissue of the left nasal cavity and the respective maxillary and ethmoidal sinuses, following the identification of progressive worsening of necrosis in a new magnetic resonance imaging study. Despite this, the patient continued to show imaging deterioration with increased filling of the left ethmoidal sinuses and abscesses. Repeated anatomopathological examinations of the nasal biopsy maintained the presence of fungal hyphae. After another multidisciplinary discussion, given the risks associated with aggressive further debridement, it was decided to maintain medical therapy and hyperbaric oxygen. A poor prognosis was established at this phase.

The rest of the hospitalization was concluded in an internal medicine ward in close collaboration with otolaryngologists, maxillofacial surgeons, and endocrinologists. After six weeks of dual antifungal therapy, the patient remained clinically stable, transitioning to isavuconazole monotherapy. He also completed 24 hyperbaric sessions, necessitating bilateral tympanostomy due to tympanic barotrauma. Under this strategy, he continued to show good clinical progress.

Throughout hospitalization, the management of the patient's metabolic profile was complex, requiring tight assessment by endocrinologists and multiple adjustments to the insulin schedule to achieve good glycemic control. Remaining clinically stable and having the possibility of outpatient follow-up, he was discharged after a total of 71 days of hospitalization. Despite all odds, the patient has shown a positive evolution after discharge. Currently, the patient is being followed up at our hospital, maintaining suppressive therapy with isavuconazole for over two consecutive years, with serial imaging evaluations outlining a stabilization of the tissue inflammation of the sinuses and persistence of an abscess in the left orbit, superimposed in dimensions. The gastric tube was removed, and currently, he is feeding on his own. The patient maintains suboptimal metabolic control due to poor adherence to diet despite regular follow-ups in consultation and has no complications or side effects from antifungal therapy. Subsequent regular debridement of nasal mucosa by fibroscopy has yielded an absence of fungal hyphae. Despite prolonged hospitalization and the complexity of the disease, the patient does not have major limitations in daily activities compared to his pre-admission state, despite having moderate visual acuity impairment. Likewise, there are no major external signs of facial mutilation, maintaining only a chronic ptosis of the left eye.

## Discussion

Mucormycosis is a fungal infection caused by Mucorales molds, with rhino-orbito-cranial involvement being the most common presentation. Despite its low incidence, the high mortality rate and the difficulties associated with its treatment make it a challenging disease. Inflammatory signs of the face and nasal cavities are its most common presentation. A CT scan revealing invasion of the nasal sinuses and isolation of Mucorales fungus in a biopsy confirm the diagnosis. The gold standard of treatment consists of surgical debridement and antifungal therapy.

In this case report, we presented a case of rhino-orbital mucormycosis in a diabetic patient, characterized by severe invasion of the nasal cavities and sinuses, with orbital involvement. A gold standard treatment of surgical debridement and antifungal therapy, supplemented with hyperbaric oxygen, was initiated with a good overall outcome. Examining the success of this case is important, as it may provide insights into better management of this disease.

This case contrasts with the usual course of rhino-orbital mucormycosis, where mortality can reach 60% to 90%, depending on the extent of intracranial involvement [4-7]. Although there are no convincing data in the literature supporting the use of dual antifungal therapy, it was employed in this patient with good clinical results, despite an unfavorable initial imaging evolution.

The duration of treatment is another controversial topic in the literature, with recommendations suggesting at least six weeks of intravenous antifungal medication followed by six months of oral antifungal medication. However, treatment may need to be extended in cases of poor clinical or imaging evolution, and lifelong therapy may be necessary when the immunosuppressive factor cannot be fully eliminated [8]. In the current case, we used prolonged, multi-year monotherapy with isavuconazole, resulting in suppression of disease progression without significant side effects. The decision for prolonged treatment was based on persistent poorly controlled diabetes and signs of active disease in the left orbit. Although the long-term side effects of such an extended treatment course are uncertain due to the lack of available literature, the positive outcome and absence of major side effects in our patient suggest that isavuconazole might be a safe option for lifelong therapy.

The relevance of conducting a new biopsy to distinguish between cure and chronic mucormycosis is currently under discussion, as it would help determine whether antifungal therapy can be safely discontinued. Further debridement, which could involve extending to the orbit to eliminate a potential active abscess, is also under consideration. However, given the expected severe mutilation from such surgery and the lack of substantial evidence that it reduces mortality, this approach may not be the best option at this stage.

Although evidence for hyperbaric oxygen is limited, it is recommended as a supplementary treatment for these patients. Hyperoxygenation of tissues may have fungistatic properties and promote healing [9]. In our case report, we cannot rule out a substantial effect of hyperbaric oxygen, though further studies are needed to draw definitive conclusions about the efficacy of this treatment. Moreover, it is essential to remain vigilant about potential side effects, such as barotrauma, which was observed in our patient.

A multidisciplinary approach was crucial for achieving a successful outcome. As surgical treatment is the gold standard of therapy, the roles of maxillofacial surgeons and otolaryngologists were vital for initial debridement of infected tissue and subsequent regular follow-ups, which showed a positive outcome. Endocrinologists also play a key role in managing diabetes, as metabolic control has been shown to be a critical pillar of successful treatment. The placement of an endogastric feeding tube by gastroenterologists ensured proper healing of the hard palate.

Mucormycosis continues to pose a significant challenge due to its rapid progression, diagnostic difficulties, and high mortality rates. This case emphasizes the need for proactive measures to reduce risk, particularly in high-risk groups such as individuals with diabetes. Optimizing glycemic control, avoiding unnecessary immunosuppression, and maintaining vigilance for early signs of infection are essential steps to improve outcomes.

## Conclusions

This case highlights the importance of a comprehensive and multidisciplinary approach in managing rhino-orbital mucormycosis. Despite the typically high mortality rate associated with this condition, the patient’s survival and stabilization underscore the potential of aggressive surgical debridement combined with tailored antifungal therapy and other adjunctive treatments such as hyperbaric oxygen.

Furthermore, the integration of a multidisciplinary team, including surgeons and endocrinologists, was pivotal in addressing the complex needs of the patient. As mucormycosis remains a formidable adversary, this case serves as a reminder of the necessity for continued research and awareness to enhance diagnostic tools and therapeutic strategies for this devastating condition.

## References

[1] Kauffman CA, Malani AN (2007). Zygomycosis: an emerging fungal infection with new options for management. Curr Infect Dis Rep.

[2] GA GR, WE AM (1961). Studies of opportunistic fungi. I. Inhibition of Rhizopus oryzae by human serum. Am J Med Sci.

[3] (2024). Mucormycosis. https://www.who.int/india/home/emergencies/coronavirus-disease-%28covid-19%29/mucormycosis.

[4] Roden MM, Zaoutis TE, Buchanan WL (2005). Epidemiology and outcome of zygomycosis: a review of 929 reported cases. Clin Infect Dis.

[5] Choksi T, Agrawal A, Date P (2022). Cumulative mortality and factors associated with outcomes of mucormycosis after COVID-19 at a multispecialty tertiary care center in India. JAMA Ophthalmol.

[6] Strasser MD, Kennedy RJ, Adam RD (1996). Rhinocerebral mucormycosis. Therapy with amphotericin B lipid complex. Arch Intern Med.

[7] Kontoyiannis DP, Lewis RE (2011). How I treat mucormycosis. Blood.

[8] Memar MY, Yekani M, Alizadeh N, Baghi HB (2019). Hyperbaric oxygen therapy: antimicrobial mechanisms and clinical application for infections. Biomed Pharmacother.

[9] Jeong W, Keighley C, Wolfe R, Lee WL, Slavin MA, Kong DC, Chen SC (2019). The epidemiology and clinical manifestations of mucormycosis: a systematic review and meta-analysis of case reports. Clin Microbiol Infect.

